# Supplementary material for: The decoration of specialized metabolites influences stylar development
Source: eLife. 2018 Oct 5;7:e38611. doi: 10.7554/eLife.38611 (PMC6192696; doi:10.7554/eLife.38611)
Supplement: Supplementary file 1. [file elife-38611-supp1.docx]

**Supplementary file 1.** **Gene accession numbers used in this study**

| **Name** | **Species** | **Accession number** |
| --- | --- | --- |
| NtMaT1 | *Nicotiana tabacum* | BAD93691 |
| NbMaT1 | *Nicotiana benthamiana* | KY563646 |
| Lp2MaT1 | *Lamium purpereum* | AAS77404 |
| Vh3MaT1 | *Verbena × hybrid* | AAS77402 |
| Pf5MaT | *Perilla frutescens* | AAL50565 |
| Ss5MaT1 | *Salvia splendens* | AAL50566 |
| Ss5MaT2 | *Salvia splendens* | AAR26385 |
| Dv3MaT | *Dahlia variabilis* | AAO12206 |
| Sc3MaT | *Senecio cruentus* | AAO38058 |
| Dm3MaT1 | *Chrysanthemum x morifolium* | AAQ63615 |
| Dm3MaT2 | *Chrysanthemum x morifolium* | AAQ63616 |
| GmIF7MaT | *Glycine max* | BAF73621 |
| At5MaT | *Arabidopsis thaliana* | NP_189600 |
| AtPMaT1 | *Arabidopsis thaliana* | AAK96528 |
| AtPMaT2 | *Arabidopsis thaliana* | NP_189609 |
| MtMaT3 | *Medicago truncatula* | ABY91222 |
| MtMaT1 | *Medicago truncatula* | ABY91220 |
| MtMaT2 | *Medicago truncatula* | ABY91221 |
| MtMaT4 | *Medicago truncatula* | ADV04046 |
| MtMaT5 | *Medicago truncatula* | ADV04047 |
| MtMaT6 | *Medicago truncatula* | ADV04048 |
