# Supplementary material for: The decoration of specialized metabolites influences stylar development
Source: eLife. 2018 Oct 5;7:e38611. doi: 10.7554/eLife.38611 (PMC6192696; doi:10.7554/eLife.38611)
Supplement: Supplementary file 2. [file elife-38611-supp2.docx]

**Supplementary file 2. The transcripts per million (TPM) values of DTGs biosynthesis pathway genes, *NaStyle2.1* and *NaYUC-like*, from RNAseq analysis.**

| Gene_id | LEC | LET | STT | COE | STI | SNP | STO | STS | NEC | ANT | OVA | PED | COL | OFL | FLB | ROT |
| --- | --- | --- | --- | --- | --- | --- | --- | --- | --- | --- | --- | --- | --- | --- | --- | --- |
| *NaMaT1* | **26** | **782** | **77** | **14** | **1.6** | **5.2** | **7.1** | **7.9** | **19** | **5.3** | **43** | **3.5** | **5.1** | **9.1** | **100** | **0.3** |
| *NaMaT2* | **0.1** | **3.63** | **9.4** | **0.1** | **0** | **0.5** | **0.7** | **0.3** | **0** | **0.1** | **0.2** | **1.2** | **0** | **0** | **0.3** | **0** |
| *NaMaT3* | **17** | **24.8** | **1.9** | **0.7** | **0.4** | **0.4** | **0.1** | **0.4** | **0.5** | **0.1** | **0.2** | **0.7** | **0.7** | **1** | **0.2** | **0.1** |
| *NIATv7_g21823* | **0.1** | **0.18** | **34** | **535** | **214** | **301** | **190** | **234** | **296** | **34** | **357** | **79** | **657** | **541** | **29** | **0.1** |
| *NIATv7_g39356* | **0.6** | **5.65** | **4.6** | **121** | **85** | **150** | **132** | **138** | **304** | **53** | **59** | **4.1** | **172** | **185** | **25** | **0.1** |
| *NaGGPPS* | **81** | **571** | **39** | **26** | **11** | **5.9** | **3.7** | **4.9** | **23** | **2.6** | **55** | **26** | **7.9** | **13** | **192** | **7.8** |
| *NaGLS* | **232** | **2784** | **1.8** | **1** | **1.1** | **0** | **0.1** | **0** | **9.1** | **0.6** | **19** | **2.7** | **0.2** | **0.4** | **315** | **0.3** |
| *NaGT1* | **563** | **2577** | **50** | **55** | **5.4** | **0.1** | **0.3** | **0.4** | **10** | **3.9** | **48** | **30** | **0.4** | **0.5** | **579** | **2** |
| *NaGT2* | **3.3** | **36.8** | **0.9** | **5.8** | **0.6** | **1.3** | **0.4** | **0.5** | **3.5** | **0.6** | **4** | **3.5** | **1.4** | **2.7** | **6.2** | **17** |
| *NaRT1* | **115** | **1480** | **2.9** | **142** | **2.8** | **0** | **0.2** | **0.1** | **59** | **8.6** | **149** | **10** | **17** | **16** | **710** | **0.3** |
| *NaStyle2.1* | **1.1** | **0.68** | **0.9** | **36** | **0.7** | **0** | **0** | **0** | **2.8** | **3.6** | **8.4** | **15** | **0** | **2.7** | **32** | **0.5** |
| *NaYUC-like-1* | **0.4** | **0.25** | **0** | **0.1** | **0** | **0** | **0** | **0** | **0** | **0** | **0** | **0** | **0** | **0.2** | **0.1** | **3** |
| *NaYUC-like-2* | **2.9** | **1.28** | **1.5** | **0.8** | **1.4** | **11** | **53** | **49** | **0.4** | **0.3** | **0.6** | **1.2** | **1.6** | **0.8** | **0.6** | **0.6** |
| *NaYUC-like-3* | **0** | **0.5** | **0** | **0** | **0** | **0.1** | **0** | **0** | **0** | **0** | **0** | **0** | **0** | **0** | **0.1** | **0.2** |
| *NaYUC-like-4* | **0.3** | **0.23** | **0** | **26** | **25** | **3.8** | **1.8** | **2.2** | **2** | **9.5** | **12** | **13** | **5.4** | **4.7** | **18** | **3.9** |
| *NaYUC-like-5* | **0** | **0.13** | **0.6** | **4.1** | **0.7** | **0** | **0** | **0** | **2.3** | **0.8** | **6.7** | **0.6** | **0.1** | **4.4** | **0.3** | **0.3** |
| *NaYUC-like-6* | **0.1** | **0.31** | **0.1** | **0** | **0.1** | **0** | **0.1** | **0** | **0.7** | **0.1** | **2.6** | **1.5** | **0** | **0.3** | **0.8** | **0** |
| *NaYUC-like-7* | **0.6** | **0.04** | **0** | **1.7** | **2.9** | **1.4** | **0.6** | **1.4** | **0** | **1.4** | **0** | **0.1** | **7.7** | **3.7** | **0.1** | **0.7** |
| *NaYUC-like-8* | **0.9** | **0.47** | **0.2** | **0.5** | **0.3** | **0.1** | **0** | **0.1** | **0** | **0.4** | **0.2** | **0** | **0.9** | **0.4** | **0.7** | **2.7** |
| *NaYUC-like-9* | **0** | **0** | **0** | **0** | **0** | **0** | **0** | **0** | **0.3** | **0** | **1.5** | **0** | **0** | **0** | **0.1** | **0** |
