# Supplementary material for: The decoration of specialized metabolites influences stylar development
Source: eLife. 2018 Oct 5;7:e38611. doi: 10.7554/eLife.38611 (PMC6192696; doi:10.7554/eLife.38611)
Supplement: Supplementary file 3. [file elife-38611-supp3.docx]

**Supplementary file 3.** **DNA primers used in this study**

| **Name** | **Sequence (5’-3’)** | **Purpose** | |
| --- | --- | --- | --- |
| MaT1-c-F | TGAAAAGCCAATTACCATTGTGT | Amplify coding sequence | |
| MaT1-c-R | TGCAGCTACAATTTTGAAGTGTT | Amplify coding sequence | |
| MaT1-e-F | CACCATGGCTTCTCTGATTGTACAATG | pENTR | |
| MaT1-e-R | GAGAAAACTTAACCCATGATTGAA | pENTR | |
| MaT1-v-F | GCGGCGGTCGACTTCGCTGGTAATGTCATTTGTC | VIGS construct | |
| MaT1-v-R | GCGGCGGGATCCTTTTCTCAATTGAGGAACAAAATG | VIGS construct | |
| MaT1-q-F | CTGATTTTGGTTGGGGGAGG | RT-qPCR | |
| MaT1-q-R | TGGACAAAGACAAGCCAATCTCT | RT-qPCR | |
| MaT2-c-F | ATGGCGTCTGTGATTGAGCA | Amplify coding sequence | |
| MaT2-c-R | AGTGAACGGAGGGAGTACCA | Amplify coding sequence | |
| MaT2-e-F | CACCATGGCGTCTGTGATTGAG | pENTR | |
| MaT2-e-R | CAGAAAGCTAAGCCCGTG | pENTR | |
| MaT2-q-F | GGTAGGGCTGAAAAACTAGAGTTTGTTTCTATTG | RT-qPCR | |
| MaT2-q-R | AGGCAAAGACAAGCCAATCTCCAA | RT-qPCR | |
| MaT3-c-F | AGGGCAGAAAAATTAGAGTTCGT | Amplify coding sequence | |
| MaT3-c-R | CTCTAGATCTCCATCTGAGTCTTTGGACTTACTAA | Amplify coding sequence | |
| MaT3-e-F | CACCATGGTGTCTCTGATTGAGAAATGT | pENTR | |
| MaT3-e-R | CAGAAAGCTAAGCCCGTGA | pENTR | |
| MaT3-q-F | AGGGCAGAAAAATTAGAGTTCGTTTCCATTG | RT-qPCR | |
| MaT3-q-R | CTCTAGATCTCCATCTGAGTCTTTGGACTTACTAA | RT-qPCR | |
| NIATv7_g21823-c-F | ACTTTGTAACTTAAATCTTATAATGGCTT | Amplify coding sequence | |
| NIATv7_g21823-c-R | GACTAGTACTGTCAAAGCATCCAA | Amplify coding sequence | |
| NIATv7_g21823-e-F | ATGGCTTCTGTGATTGAGC | pENTR | |
| NIATv7_g21823-e-R | ttgtcgatagaaacgaactctaac | pENTR | |
| NIATv7_g39356-c-F | AGCTACAATGGCGTCTGTGA | Amplify coding sequence | |
| NIATv7_g39356-c-R | TCCACCAGAACACTTAAAATGCAA | Amplify coding sequence | |
| NIATv7_g39356-e-F | CACCATGGCGTCTGTGATTGAG | pENTR | |
| NIATv7_g39356-e-R | CAGAAAGCTAAGCCCGTG | pENTR | |
| NaGGPPS-q-F | GATGATCCACACTATGTCCCTC | RT-qPCR | |
| NaGGPPS-q-R | CTCGCCGTAGACTTTATGGT | RT-qPCR | |
| NaGLS-q-F | TCTGGCCCTATGTTTGAGAG | RT-qPCR | |
| NaGLS-q-R | CACCCACATTCCATTTCTTGAG | RT-qPCR | |
| NIATv7_g19298-q-F | CGTGCCTGCAAAAATGGGAT | RT-qPCR | |
| NIATv7_ g19298-q-R | GGACAGCAGTCATGGCCAAA | RT-qPCR | |
| NIATv7_ g25593-q-F | ACCTCGCCAAGCTTAAGACG | RT-qPCR | |
| NIATv7_ g25593-q-R | GCGTCTTGTCCCCATAATGC | RT-qPCR | |
| NIATv7_g39188-q-F | GCTAGCCATGCATCCACAGT | RT-qPCR |  |
| NIATv7_g39188-q-R | TGCAACGATCGGAGGGTAAA | RT-qPCR |  |
| NaYUC-like 2-q-F | CCTGTTATTATTGGAGCTGGTC | RT-qPCR | |
| NaYUC-like 2-q-R | TAGCAGTGTGGACCGACTC | RT-qPCR | |
| IF5a-q-F | gtcggacgaagaacaccatt | RT-qPCR | |
| IF5a-q-R | cacatcacagttgtgggagg | RT-qPCR | |
